# The impact of COVID-19 pandemic on the healthcare of premature babies

**DOI:** 10.18332/ejm/122385

**Published:** 2020-05-21

**Authors:** Eleni Vavouraki

**Affiliations:** 1 European Foundation for the Care of Newborn Infants (EFCNI), Munich, Germany; 2 "Ilitominon" For the Care of Premature Newborns, Athens, Greece

**Keywords:** premature babies, breastfeeding, NICU, parental bonding, COVID-19, parent-newborn separation

**Dear Editor,**

As the COVID-19 pandemic reaches every country around the world, there are concerns raised by Parent Organisations about the severe consequences that the pandemic may have for the health of premature and sick babies.

In an attempt to control the spread of the virus, the vast majority of NICUs have limited parental access, and the restrictions differ not only between countries but also between hospitals within each country. Moreover, no one has provided parents with an evidencebased explanation for the necessity of keeping parents out of the NICUs while most of the Paediatric Units or Hospitals continue to welcome the parents of their patients.

Admittedly, the situation we are facing is new, and it may be too early to have an evidence-based strategy. Separation, though, also has a risk that should not be overlooked for any reason. Early separation is harmful to both newborn infants and their parents since it disrupts the biological and emotional bonding that has developed already during gestation^1,2^. Limiting the time of access to a few minutes or stopping the access to their babies will have significant health and mental consequences for the parents and the baby, and this is an impact of the pandemic that must be taken very seriously.

Another severe consequence is that by keeping mothers isolated from their babies, a trend of reduction in breastfeeding may develop, as it is not possible to apply supportive techniques in the NICU such as skin-to-skin contact and continuous midwifery counselling. Even more worrying is that this trend has emerged despite the recommendations^3^ of the World Health Organization (WHO) that during COVID-19 pandemic healthcare providers should ‘enable mothers and infants to remain together and practice skin-to-skin contact, and rooming-in throughout the day and night, especially straight after birth during the establishment of breastfeeding, whether or not the mother or child has suspected, probable, or confirmed COVID-19’.

Importantly, follow-up appointments, therapy and psychological support services have stopped in many places across the world as clinics and rehabilitation centers have suspended their operations during the lockdown, causing many parents great concern for the unpredictable consequences to their child’s health^4,5^.

Parent Organisations appreciate health professionals’ work and engagement in fighting the pandemic, the health of whom must be equally protected from the risks of COVID-19 exposure. It is understood that in many cases, the restricting regulations were made based on the available human resources and the adequacy of materials, such as masks, gloves, and uniforms. However, everyone’s health can and should be protected by such decisions that will not cause long-term consequences to children’s health.

Urgent action must be taken to protect parents and newborns, to prevent any collateral damage to their health. We are confident that we will once again have midwives by our side as advocates of family-centred care and breastfeeding, for healthy families and children. Together we can ensure the provision of high-level maternal and neonatal health care, even during a challenging period, such as the one we are going through now.
